# Epigenetic alterations leading to TMPRSS4 promoter hypomethylation and protein overexpression predict poor prognosis in squamous lung cancer patients

**DOI:** 10.18632/oncotarget.8045

**Published:** 2016-03-14

**Authors:** Maria Villalba, Angel Diaz-Lagares, Miriam Redrado, Arrate L. de Aberasturi, Victor Segura, Maria Elena Bodegas, Maria J. Pajares, Ruben Pio, Javier Freire, Javier Gomez-Roman, Luis M. Montuenga, Manel Esteller, Juan Sandoval, Alfonso Calvo

**Affiliations:** ^1^ Department of Histology and Pathology, School of Medicine, University of Navarra, Pamplona, Navarra, Spain; ^2^ IDISNA and Program in Solid Tumors and Biomarkers, Center for Applied Medical Research (CIMA), University of Navarra, Pamplona, Navarra, Spain; ^3^ Cancer Epigenetics and Biology Program (PEBC), Bellvitge Biomedical Research Institute (IDIBELL), L'Hospitalet, Catalonia, Spain; ^4^ IDISNA and Bioinformatics Unit, Center for Applied Medical Research (CIMA), University of Navarra, Pamplona, Navarra, Spain; ^5^ Department of Biochemistry and Genetics, School of Sciences, University of Navarra, Pamplona, Navarra, Spain; ^6^ Department of Pathology, University Hospital Marques de Valdecilla, IDIVAL, Santander, Spain; ^7^ Department of Personalized Medicine, Epigenomics Unit, Medical Research Institute La Fe, Valencia, Spain

**Keywords:** TMPRSS4, epigenetics, promoter hypomethylation, squamous cell carcinoma, prognosis

## Abstract

Non-small cell lung cancer (NSCLC) is the leading cause of cancer-related death worldwide, which highlights the need of innovative therapeutic options. Although targeted therapies can be successfully used in a subset of patients with lung adenocarcinomas (ADC), they are not appropriate for patients with squamous cell carcinomas (SCC). In addition, there is an unmet need for the identification of prognostic biomarkers that can select patients at risk of relapse in early stages. Here, we have used several cohorts of NSCLC patients to analyze the prognostic value of both protein expression and DNA promoter methylation status of the prometastatic serine protease TMPRSS4. Moreover, expression and promoter methylation was evaluated in a panel of 46 lung cancer cell lines. We have demonstrated that a high TMPRSS4 expression is an independent prognostic factor in SCC. Similarly, aberrant hypomethylation in tumors, which correlates with high TMPRSS4 expression, is an independent prognostic predictor in SCC. The inverse correlation between expression and methylation status was also observed in cell lines. *In vitro* studies showed that treatment of cells lacking TMPRSS4 expression with a demethylating agent significantly increased TMPRSS4 levels. In conclusion, TMPRSS4 is a novel independent prognostic biomarker regulated by epigenetic changes in SCC and a potential therapeutic target in this tumor type, where targeted therapy is still underdeveloped.

## INTRODUCTION

Lung cancer still remains as the most common cause of cancer death worldwide and presents a 5-year relative survival of around 18% [1, 2]. Among the different tumor subtypes, non-small cell lung cancer (NSCLC) is the most common one (accounting approximately for 85% of the cases) and comprises two major histological groups: adenocarcinomas (ADC, ∼50%), and squamous cell carcinomas (SCC, ∼30%). Late detection is a key factor related to its high mortality rate. In fact, it has been shown that earlier detection in screening programs mediated by spiral computed tomography reduces lung cancer deaths [3]. However, current therapeutic regimes are not sufficiently effective to significantly increase long term survival. For most patients with early NSCLC, surgical resection is the appropriate therapeutic option, although a considerable percentage of them (∼30-70%, depending on the stage) will relapse overtime [2].

The implementation of targeted therapies aimed to inhibit specific mutations is mainly beneficial for a small group of patients with advanced NSCLC displaying adenocarcinoma histology [4], but not for patients with SCC [5]. Thus, clinical trials using targeted therapies against potential driver alterations have not been successful in SCC, and chemotherapy, or more recently immunotherapy [6], remains as the current option for these patients. Therefore, it seems clear that a better understanding of the biology of SCC is critical to identify effective therapeutic options.

Genetic abnormalities associated with SCC that are under-represented in ADC include PI3KCA, SOX2, and FGFR1 amplification, PTEN loss, and DDR2 and TP53 mutations [5]. Although patients with SCC cannot be currently selected for targeted therapy based on their molecular tumor profile, the aforementioned alterations and emerging targets constitute a window of opportunity for new therapies.

In a previous study, we demonstrated that TMPRSS4 was highly upregulated in NSCLC, particularly in SCC, in comparison to non-malignant lung [7]. Moreover, we showed that increased TMPRSS4 mRNA levels were associated with poor prognosis in SCC patients [7]. TMPRSS4 has also been found upregulated in colon, pancreas, breast, cervix, thyroid and liver tumors [8, 9, 10, 11]. This protein increases tumor growth and metastasis and induces the acquisition of epithelial to mesenchymal transition (EMT) [12, 13] and cancer stem cell (CSC) phenotypes [14].

Epigenetic control of gene expression acts as a switch to either induce or repress the transcriptional activity of multiple genes implicated in cancer development. Specific epigenetic mechanisms have been identified as responsible to regulate the expression of certain EMT-related genes [15, 16]. One of these epigenetic mechanisms is DNA methylation, which consists in the addition of a methyl group to the 5′ carbon of cytosine within cytosine-guanine dinucleotides (CpGs). Alterations in methylation patterns impair the transcriptional balance of cells and contribute to pathological conditions, including cancer initiation and progression [15].

In this study, we have evaluated the prognostic value of both TMPRSS4 protein levels and promoter methylation status in NSCLC patients. *In vitro* experiments were performed as well to demonstrate that TMPRSS4 expression is regulated by promoter methylation. We have found that TMPRSS4 overexpression correlates with poor prognosis in NSCLC patients with squamous histology. Abnormal promoter hypomethylation was found in tissue samples from patients but not in non-malignant tissues and was inversely correlated with TMPRSS4 expression. Moreover, hypomethylation was associated with reduced relapse free survival in patients.

## RESULTS

### High TMPRSS4 protein expression is significantly associated with reduced RFS and OS in NSCLC patients with squamous histology

**Figure 1:**
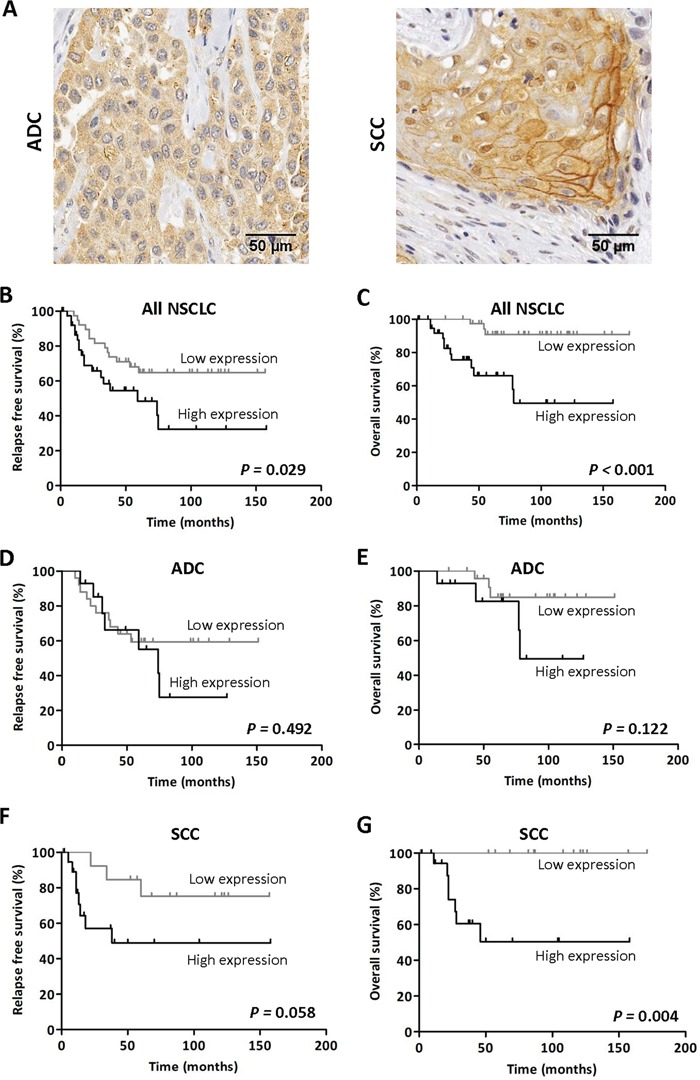
High TMPRSS4 protein expression correlates with poor prognosis **A.** Immunohistochemical staining for TMPRSS4 protein in adenocarcinoma (ADC, left panel) and squamous cell carcinoma (SCC, right panel). Strong membrane immunoreactivity was observed in some SCC samples. **B-C.** Kaplan-Meier curves in the whole set of patients showing that high TMPRSS4 levels are significantly associated with reduced relapse free survival (RFS) and overall survival (OS). **D-E.** In patients with ADC histology no statistical differences were observed for either RFS or OS in relation to TMPRSS4 protein levels. **F-G.** In SCC, high TMPRSS4 levels were very significantly associated with lower OS (p=0.004), and were borderline for RFS (p=0.058). Differences between groups were assessed by the logRank test.

We first performed an immunohistochemical study on tissue microarrays (TMAs) containing NSCLC (n=79, stages I-II) and matched non-malignant (n=66) samples. The clinical and pathological characteristics of the NSCLC patients are shown in Supplementary Table 1. In non-malignant lung samples, immunostaining was very low and only observed in some type II pneumocytes (Supplementary Figure 1A) and bronchiolar epithelial cells. In malignant specimens, the signal was found in tumor cells in both adenocarcinomas (ADC) and squamous cell carcinomas (SCC) (Figure 1A). In some SCC, immunostaining could also be detected in the plasma membrane of tumor cells. Tumors showed a very significant increase in H-score (p<0.001) in comparison with non-malignant samples (Supplementary Figure 1B). NSCLC patients were dichotomized into two groups: high (n=40) and low (n=39) TMPRSS4 expression, according to the median value of the H-score.

We then evaluated the relationship between TMPRSS4 protein expression and clinicopathological characteristics of the patients. No association between TMPRSS4 expression and any of the clinicopathological characteristics analyzed was found except for the histological type (p=0.017; Supplementary Table 2). Remarkably, the frequency of tumors with high TMPRSS4 was significantly higher in SCC than in ADC tumors.

Regarding prognosis, patients with high levels of TMPRSS4 showed significantly shorter relapse free survival (RFS, p=0.029) and overall survival (OS, p<0.001) than those with low levels (Figure 1B-1C). We next stratified the patients according to their histological tumor type. In ADC patients, no statistical association was found between TMPRSS4 levels and either RFS (p=0.492) or OS (p=0.122) (Figure 1D-1E). On the contrary, in SCC patients, high TMPRSS4 levels were significantly associated with reduced OS (p=0.004) and were close to statistical significance for RFS (p=0.058) (Figure 1F-1G).

When considering only the cases corresponding to stage I patients (n=52), we found that high TMPRSS4 protein expression was significantly correlated with worse OS (p=0.001) and borderline for RFS (p=0.058) (Supplementary Figure 1C-1D). Similar results were obtained when the analysis was performed exclusively in stage II patients (n=27) (Supplementary Figure 1E-1F). In these latter sets of patients we did not perform the analysis after stratification by histological types due to the low number of cases.

To rule out the possibility that post-surgery treatment with chemotherapy or radiotherapy could influence the results on survival, we performed logRank tests excluding patients treated with post-surgery adjuvant therapy. As shown in Supplementary Figure 1G, results were similar to those found for the whole cohort of patients, indicating that high TMPRSS4 levels were significantly associated with reduced RFS and OS in SCC and all NSCLC patients. In this case, a significant association (p=0.023) between high TMPRSS4 expression and OS was also observed for ADC.

We next evaluated if TMPRSS4 could be considered as an independent prognostic factor in NSCLC using Cox regression analyses (Table 1). In the univariate analysis, high TMPRSS4 levels were significantly associated with reduced RFS (HR=2.2; 95%CI: 1.1-4.5; p=0.033) and OS (HR=7.1; 95%CI: 2.0-25.6; p=0.003). Age, gender, smoking status, post-surgery radio or chemotherapy, histology, grade and stage were not found as predictors of either RFS or OS. For the multivariate analysis, significant variables as well as variables considered clinically relevant and close to statistical significance were included. Multivariate analysis in the whole cohort of patients indicated that TMPRSS4 was an independent prognostic factor for both RFS (HR: 2.3; 95%CI: 1.1-4.8; p=0.020) and OS (HR: 6.0; 95%CI: 1.6-2.2; p=0.007). Stage was also found as a significant prognostic factor of OS (HR: 3.1; 95%CI 1.1-9.0; p=0.041). We then conducted univariate and multivariate analyses after stratification of patients according to histological subtypes. Table 2 shows that, in the univariate analysis performed in SCC, high TMPRSS4 was found to be associated with reduced RFS (HR: 5.3; 95%CI: 1.1-25.3; p=0.036). In multivariate analysis we confirmed this association in SCC (HR: 5.4; 95%CI: 1.1-25.7; p=0.035). Regarding OS in SCC, univariate and multivariate analyses could not be performed, since data did not fulfill the criteria of proportional hazards (due to the lack of events in the group of patients with low TMPRSS4 expression; see Figure 1G). For ADC, no statistical association between TMPRSS4 protein expression and either RFS or OS was observed (Table 2).

**Table 1:** Univariate and multivariate Cox regression analysis to study the effect of TMPRSS4 expression on RFS and OS in NSCLC patients

	RFS					
	Univariate analysis			Multivariate analysis		
	HR	95% CI	*P*	HR	95% CI	*P*
**Age**						
< 65						
≥ 65	1,359	0.671-2.752	0.395			
**Gender**						
Female						
Male	0.759	0.311-1.854	0.545			
**Smoking Status**						
Never smoker						
Former smoker	0.487	0.179-1.326	0.159			
Current smoker	0.990	0.330-2.966	0.985			
Radiotherapy*						
No						
Yes	1,954	0.683-5.594	0.212			
Chemotherapy*						
No						
Yes	1,553	0.757-3.188	0.230			
**Histology**						
ADC						
SCC	0.924	0.433-1.975	0.839			
Others	2,718	0.781-9.454	0.116			
**Grade**						
WD/MD						
PD	1,300	0.626-2.698	0.481			
**Stage**						
I						
II	1,730	0.845-3.540	0.134	1,933	0.956-3.908	0.066
**TMPRSS4**						
Low						
High	2,187	1.064-4.494	**0.033**	2,335	1.142-4.775	**0.020**
**Age**						
< 65						
≥ 65	1,969	0.701-5.536	0.199			
**Gender**						
Female						
Male	2,579	0.339-19.637	0.360			
**Smoking Status**						
Never smoker						
Former smoker	1,701	0.219-13.187	0.611			
Current smoker	1,458	0.151-14.044	0.744			
Radiotherapy*						
No						
Yes	2,742	0.766-9.821	0.121			
Chemotherapy*						
No						
Yes	0.866	0.275-2.724	0.805			
**Histology**						
ADC						
SCC	1,412	0.494-4.034	0.519			
Others	2,108	0.253-17.557	0.490			
**Grade**						
WD/MD						
PD	2,831	0.948-8.460	0.062	2,102	0.696-6.347	0.188
**Stage**						
I						
II	2,646	0.957-7.315	0.061	3,072	1.048-9.007	**0.041**
**TMPRSS4**						
Low						
High	7,136	1.991-25.574	**0.003**	5,970	1.634-2.181	**0.007**

WD = well differentiated; MD = moderately differentiated; PD = poorly differentiated.

* indicates that radiotherapy or chemotherapy were applied post-therapy.

**Table 2:** Univariate and multivariate Cox regression analyses to study the effect of TMPRSS4 expression in ADC and SCC patients

	ADC								
	RFS			OS					
	Univariate analysis			Univariate analysis			Multivariate analysis		
	HR	95% CI	*P*	HR	95% CI	*P*	HR	95% CI	*P*
**Age**									
< 65									
≥ 65	0.876	(0.333-2.305)	0.788	0.429	(0.082-2.246)	0.316			
**Gender**									
Female									
Male	0.556	(0.194-1.596)	0.275	1,526	(0.183-1.272)	0.696			
**Smoking Status**									
Never smoker									
Former smoker	0.504	(0.154-1.649)	0.257	1,276	(0.149-1.096)	0.824			
Current smoker	1,481	(0.366-5.991)	0.582	1,371	(0.086-2.193)	0.824			
Radiotherapy*									
No									
Yes	2,253	(0.506-10.031)	0.286	1,957	(0.233-16.410)	0.536			
Chemotherapy*									
No									
Yes	1,552	(0.585-4.118)	0.377	0.764	(0.148-3.945)	0.748			
**Grade**									
WD/MD									
PD	1,991	(0.719-5.514)	0.185	10,190	(1.188-87.416)	**0.034**	6,808	(0.723-64.083)	0.094
**Stage**									
I									
II	1,996	(0.763-5.217)	0.159	2,576	(0.575-11.530)	0.216	2,423	(0.385-15.249)	0.346
**TMPRSS4**									
Low									
High	1,403	(0.532-3.696)	0.494	3,078	(0.687-13.801)	0.142	1,875	(0.343-10.254)	0.469

	SCC					
	RFS					
	Univariate analysis			Multivariate analysis		
	HR	95% CI	*P*	HR	95% CI	*P*
**Age**						
< 65						
≥ 65	1,794	(0.519-6.197)	0.355			
**Gender**						
Female						
Male	‡	‡	‡			
**Smoking Status**						
Never smoker						
Former smoker						
Current smoker	1,747	(0.529-5.766)	0.360			
Radiotherapy*						
No						
Yes	1,743	(0.220-13.826)	0.599			
Chemotherapy*						
No						
Yes	0.556	(0.071-4.346)	0.576			
**Grade**						
WD/MD						
PD	0.742	(0.217-2.541)	0.635			
**Stage**						
I						
II	1,200	(0.317-4.541)	0.788	1,288	(0.336-4.938)	0.712
**TMPRSS4**						
Low						
High	5,317	(1.117-25.301)	**0.036**	5,381	(1.127-25.694)	**0.035**

WD = well differentiated; MD = moderately differentiated; PD = poorly differentiated

* indicates that radiotherapy or chemotherapy were applied post-therapy.

‡ indicates that the test was not applicable because there were only two females in that group.

All these data indicate that TMPRSS4 is an independent prognostic factor at early stages in NSCLC, associated with poor prognosis in SCC patients.

### Aberrant hypomethylation of TMPRSS4 promoter in NSCLC

**Figure 2:**
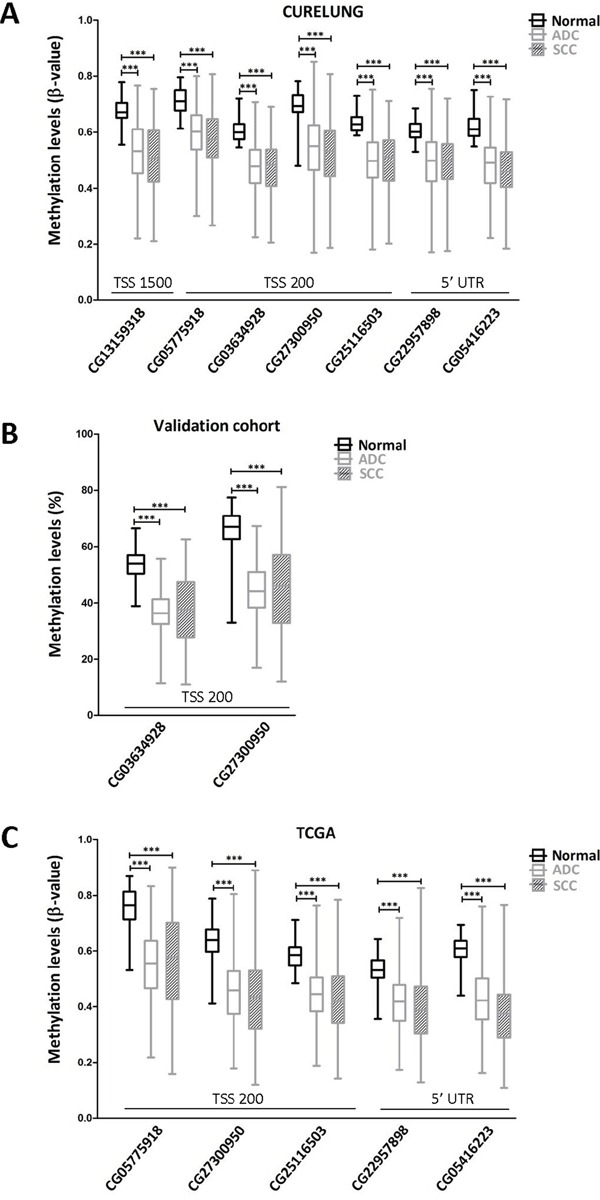
TMPRSS4 promoter hypomethylation is consistently found in NSCLC patients in comparison with their non-malignant counterparts **A.** Seven CpGs were analyzed using the 450k methylation array in the CURELUNG cohort. All CpGs showed hypomethylation in both ADC and SCC in comparison with non-malignant lung tissue. **B.** Pyrosequencing in the validation cohort of two consecutive CpGs located at the TSS200 region showed a similar hypomethylation pattern in both ADC and SCC samples. **C.** Data from TCGA, where probes CG13159318 and CG03634928 were not available, confirmed a very significant reduction in TMPRSS4 promoter methylation levels in malignant samples.

In order to ascertain the possible cause of TMPRSS4 overexpression in lung cancer patients, we evaluated through bioinformatic analyses using public databases (COSMIC, CCLE, IGDB.NSCLC) possible genetic alterations (gene amplification, mutations, rearrangements) of TMPRSS4 in NSCLC. Analysis showed that these changes were very infrequent and unlikely to explain TMPRSS4 upregulation (data not shown). We then hypothesized that increased TMPRSS4 expression could be due to epigenetic modifications. The TMPRSS4 promoter does not contain a canonical CpG island, but gene overexpression through hypomethylation in non-CpG islands has been previously described [17]. Based on this fact, we studied TMPRSS4 promoter methylation status in NSCLC and normal samples using the 450k methylation array (FP7 CURELUNG discovery cohort). We analyzed methylation levels (Δ-values) of CpGs located at the following positions from the Transcription Start Site (TSS): CpG −268 bp (CG13159318 probe in the 450k methylation array), CpG −172 bp (CG05775918), CpG −116 bp (CG03634928), CpG −99 bp (CG27300950), CpG −70 bp (CG25116503), CpG +151 bp (CG22957898) and CpG +271 bp (CG05416223). The different CpGs were grouped into 3 sets according to their position: 5′ UTR, TSS200 (from the TSS to nucleotide −200) and TSS1500 (from −200 to −1500 upstream the TSS). A significant and consistent decrease in methylation levels for all the CpGs was detected in both ADC and SCC as compared to non-malignant tissues (Figure 2A).

To validate this result, DNA samples from another cohort of 88 NSCLC patients (Supplementary Table 1 for patient's characteristics) was analyzed by pyrosequencing of CpGs −116 (CG03634928) and −99 (CG27300950). We selected these CpGs for technical reasons dealing with conditions required for primer design and for being close to the TSS. As shown in Figure 2B, methylation levels in both ADC and SCC were very significantly reduced (p<0.001) with respect to those found in non-malignant lungs. To further support these results, we retrieved data from The Cancer Genome Atlas TCGA [18] and evaluated methylation levels of the TMPRSS4 promoter. In this dataset, probes CG13159318 and CG03634928 were not available. Figure 2C shows that, in agreement with the other cohorts, significant aberrant TMPRSS4 promoter hypomethylation (p<0.001) was observed for all the methylation sites studied in both ADC and SCC.

Therefore, we conclude that TMPRSS4 promoter is methylated in normal lung, whereas an abnormal hypomethylation occurs in tumors, which could constitute an oncogenic mechanism, as described for other tumor promoting genes [19].

### Hypomethylation of TMPRSS4 promoter significantly correlates with RFS in NSCLC patients

We next investigated whether promoter hypomethylation had an effect on clinical outcome. Patients were categorized based on a 50% methylation threshold. The prognostic value of loss of methylation for each of the 7 probes analyzed was assessed in the CURELUNG cohort by logRank test and multivariate Cox regression for correlation with RFS, in those patients where clinical information was available (ADC, n=155 and SCC, n=43). Data on OS was not available in this cohort.

**Figure 3:**
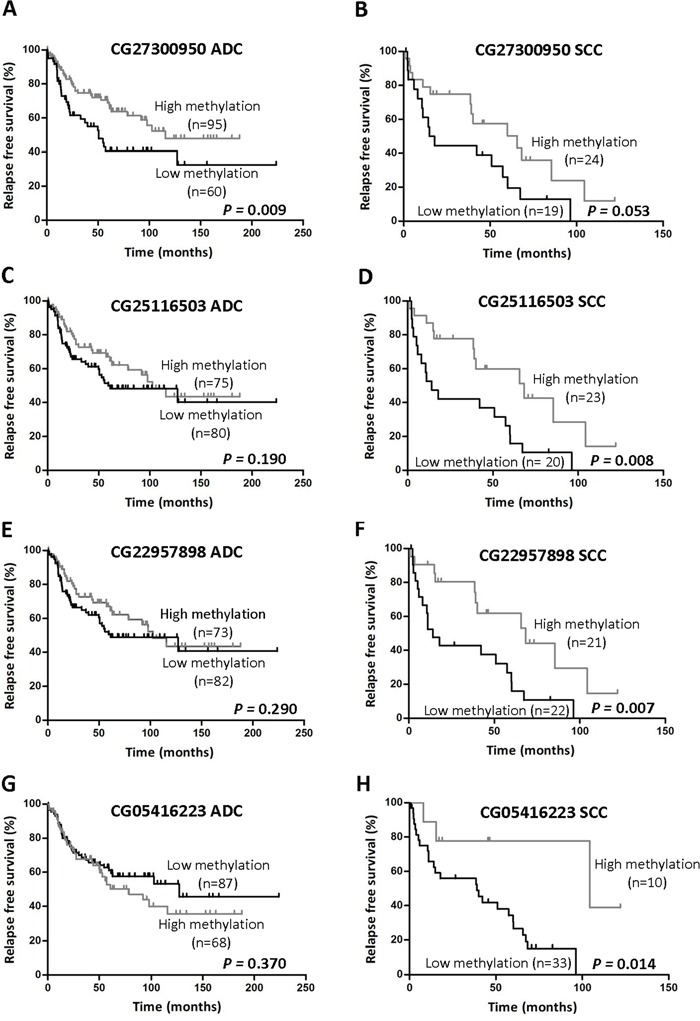
Association between TMPRSS4 promoter methylation status and RFS in NSCLC patients The previously defined 50% methylation cutoff value (β<0.5) was used to categorize patients into two groups (high and low methylation status). The following probes were depicted in ADC and SCC graphics, respectively: CG27300950 **A-B.** CG25116503 **C-D.** CG22957898 **E-F.** and CG05416223 **G-H.** Significant reduction in RFS was found for SCC patients with low TMPRSS4 promoter methylation in 3 out of 4 CpGs analyzed. Only CG27300950 was significantly different between groups in ADC and close to statistical significance in SCC.

Table 3 shows that, considering results from both logRank and multivariate analyses, there was a DNA region corresponding to probes CG03634928 to CG05416223 that contained CpGs whose hypomethylation (β<0.5) predicts poor prognosis in SCC tumors. In the multivariate analysis, hypomethylation of any of the last 4 consecutive CpGs (CG27300950 to CG05416223 probes) was an independent predictor of reduced RFS in this tumor type. In the logRank test, low methylation levels were found to be associated with worse prognosis for the following probes: CG03634928 (p=0.046), CG25116503 (p=0.008), CG22957898 (p=0.007) and CG05416223 (p=0.014). Differences for CG27300950 were close to statistical significance (p=0.053). Figure 3 illustrates Kaplan-Meier curves for the last 4 consecutive CpGs in SCC patients in comparison to those obtained in ADC.

**Table 3:** Log Rank *P* values and Cox regression analysis to study patient's RFS in relation to methylation status in CpG sites

			All samples				ADC				SCC			
			logRank	Multivariate analysis			logRank	Multivariate analysis			logRank	Multivariate analysis		
TargetID	Location*	Group	*P*	HR	95% CI	*P*	*P*	HR	95% CI	*P*	*P*	HR	95% CI	*P*
CG13159318	−268	TSS1500	**0.004**	1.28	(0.85-1.93)	0.235	**0.030**	1.30	(0.79-2.13)	0.296	0.220	1.69	(0.74-3.83)	0.211
CG05775918	−172	TSS200	**0.003**	1.42	(0.91-2.21)	0.122	**0.002**	1.69	(0.97-2.94)	0.063	0.910	1.30	(0.59-2.87)	0.520
CG03634928	−116	TSS200	0.065	1.21	(0.78-1.87)	0.389	0.230	1.13	(0.68-1.89)	0.640	**0.046**	1.87	(0.78-4.48)	0.162
CG27300950	−99	TSS200	**0.001**	1.66	(1.11-2.51)	**0.015**	**0.009**	1.42	(0.86-2.35)	0.167	0.053	3.36	(1.50-7.46)	**0.003**
CG25116503	−70	TSS200	**0.034**	1.31	(0.87-1.99)	0.198	0.190	1.06	(0.64-1.73)	0.828	**0.008**	3.23	(1.40-7.46)	**0.006**
CG22957898	+191	5′UTR	0.050	1.19	(0.78-1.81)	0.417	0.290	0.96	(0.58-1.57)	0.860	**0.007**	3.09	(1.30-7.30)	**0.011**
CG05416223	+271	5′UTR	0.570	0.98	(0.65-1.49)	0.938	0.370	0.80	(0.49-1.30)	0.368	**0.014**	4.69	(1.26-17.54)	**0.021**

* bp relative to the TSS.

Methylation patterns regarding prognosis observed for SCC were different in ADC tumors. In the multivariate analyses, TMPRSS4 promoter hypomethylation was not an independent prognostic factor for any of the CpGs analyzed in ADC (Table 3). In the logRank test, low methylation levels of CpGs corresponding to CG13159318, CG05775918 and CG27300950 probes were significantly associated with shorter RFS (Table 3 and Figure 3).

When considering all NSCLC samples, hypomethylation was an independent predictor of reduced RFS only for probe CG27300950 (p=0.015). The logRank tests showed statistical differences in the same CpGs that were obtained for ADC, plus probe CG25116503. Supplementary Figure 2A-2D illustrates Kaplan-Meier curves for all NSCLC patients corresponding to the last 4 consecutive CpGs (CG27300950 to CG05416223 probes).

These results highlight the differences in hypomethylation patterns related to prognosis found for SCC and ADC. Taking into account results from both multivariate analyses and longRank tests we conclude that loss of TMPRSS4 promoter methylation in CpGs spanning from −116 bp to +271 bp relative to the TSS (CG03634928 to CG05416223 probes) consistently predicts poor outcome in SCC.

### TMPRSS4 mRNA levels inversely correlate with the degree of promoter methylation in NSCLC patients and cell lines

We then retrieved data from both TMPRSS4 expression and promoter methylation status available at TCGA (ADC, n=454 and SCC, n=370) to perform correlation analyses with those CpGs that were localized between −116 bp to +271 bp relative to the TSS and were significantly associated with poor prognosis in the multivariate analysis.

**Figure 4:**
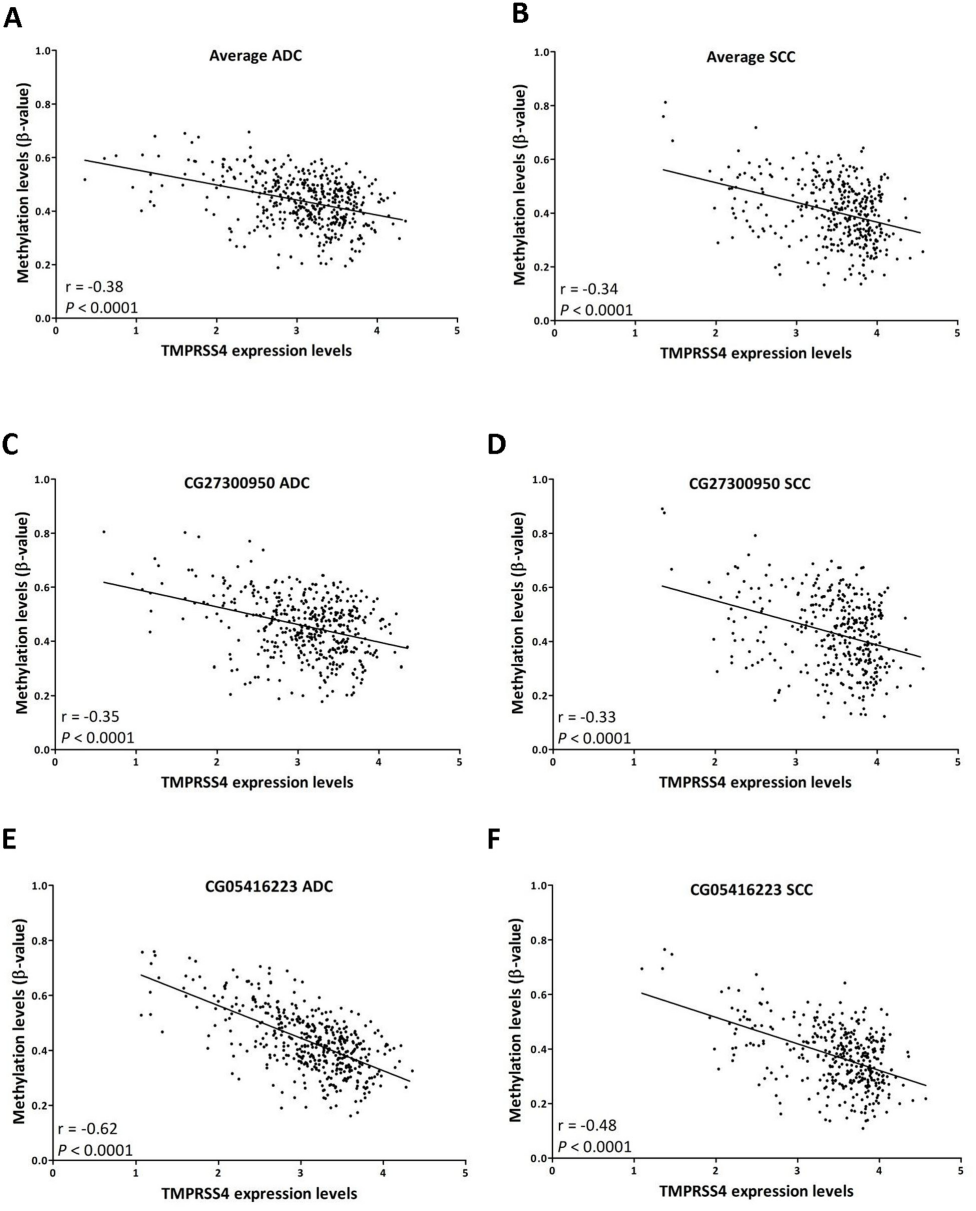
TMPRSS4 expression (RNA-Seq) inversely correlates with promoter methylation (450k methylation array) **A.** Average methylation values of CpGs corresponding to probes CG27300950 to CG05416223 (group of 4 CpGs that predicted poor prognosis in SCC in the multivariate analysis) were first considered for the analysis in ADC samples. A significant inverse correlation between methylation status and expression is observed. **B.** A similar result is found for SCC. Correlation analysis using values from individual probes follows a similar pattern. Examples of CG27300950 **C.** ADC; **D.** SCC) and CG05416223 (**E.** ADC; **F.** SCC) are shown. Data retrieved from TCGA.

Figure 4A-4B shows a significant inverse correlation between TMPRSS4 expression and methylation status for the average value of the 4 probes analyzed (r=−0.38, p<0.0001 for ADC; r=−0.34, p<0.0001 for SCC). A significant inverse correlation between expression and methylation status considering separately each of the individual probes was also observed (p<0.0001 for all of them). Figure 4C-4F shows the probes with the highest correlation values: CG27300950 (r=−0.35 for ADC; r=−033 for SCC; Figure 4C-4D) and CG05416223 (r=−0.62 for ADC; r=−048 for SCC; Figure 4E-4F).

**Figure 5:**
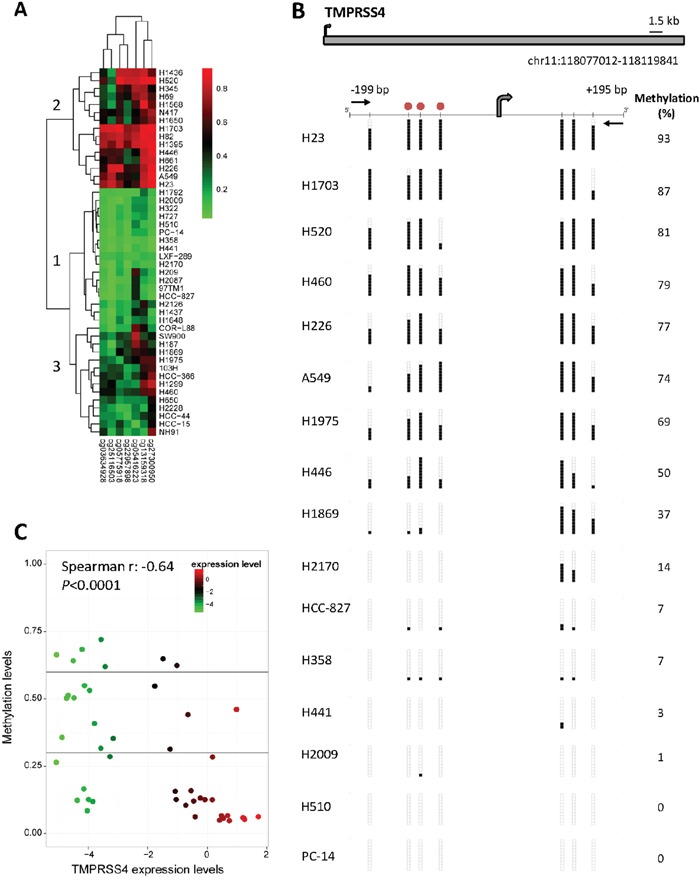
TMPRSS4 promoter methylation status in a panel of NSCLC cell lines **A.** Methyloma analysis of 46 cell lines was carried out with the 450k methylation array and hierarchical cluster analysis separated 3 groups: (1, n=17) cells with promoter hypomethylation in all or most of the 7 CpGs analyzed; (2, n=15) cells with predominant promoter methylation; and (3, n=14) cells with no clear pattern of methylation. **B.** Bisulfite genomic sequencing of TMPRSS4 promoter in human lung cancer cell lines. Location of bisulfite genomic sequencing PCR primers (black horizontal arrows), CpG dinucleotides (vertical lines) and the transcriptional start site (TSS, grey arrow) are shown. Ten single clones are represented for each sample. Presence of unmethylated or methylated CpGs is indicated by white or black squares, respectively. The CpG sites analyzed are located at −172 bp, −116 bp, −99 bp, −70 bp, +106 bp, +123 bp and +151 bp from the TSS (chr11:118.077.075), according to the current version of UCSC Genome Browser (GRCh38/hg38). Red circles represent the location of three consecutive probes (CG03634928, CG27300950 and CG25116503) detected by the 450k methylation array that correspond to −116 bp, −99 bp and −70 bp from the TSS, respectively. **C.** Graphic representation of inverse correlation between TMPRSS4 expression (evaluated by real time PCR) and methylation status (pyrosequencing) of the 46 cell lines. Spearman's correlation r=−0.64; p<0.0001.

We also evaluated both expression and promoter methylation status in a panel of 46 lung cancer cell lines. Firstly, analysis of the methylome using the 450k methylation array in these cells showed different methylation patterns of TMPRSS4 promoter (Figure 5A). Classification according to an unsupervised hierarchical cluster analysis based on methylation status separated 3 different groups: (1) cells with consistent TMPRSS4 promoter hypomethylation in all or most of the 7 CpGs analyzed; (2) cells with predominant promoter methylation; and (3), cells with a less defined pattern.

For analysis using bisulfite sequencing we selected 16 cell lines representative of the 3 different clusters previously identified in the 450k methylation array (Figure 5B). Sequencing of the CpG sites located at −172 bp (CG05775918), −116 bp (CG03634928), −99 bp (CG27300950), −70 bp (CG25116503), +106 bp, +123 bp and +151 bp (CG22957898) relative to the TSS revealed the following results: extensive methylation was observed for cells belonging to cluster 2 in the array (H23, H1703, H520, H226, A549 and H446), whereas cells with no promoter methylation were coincident with those included in cluster 1, confirming our previous results obtained with the 450k methylation array.

Methylation status was next validated by pyrosequencing of the CpGs located at −116 bp and −99 bp and results were correlated with those obtained in the array. As shown in Supplementary Figure 2E, there was a significant correlation between methylation status analyzed by both techniques (r=0.80; p<0.0001).

We then quantified TMPRSS4 mRNA levels by real time PCR in the whole set of cell lines and performed Spearman's test to correlate expression with degree of methylation (average of the 2 CpGs evaluated by pyrosequencing). Figure 5C shows that there was a statistically significant inverse correlation (r=−0.64; p<0.0001) between TMPRSS4 expression and promoter methylation.

To test whether demethylation of TMPRSS4 promoter would result in increased TMPRSS4 levels, we treated cell lines lacking TMPRSS4 expression with the demethylating agent 5-aza-2′-deoxycytidine. After 48 hours of treatment with 2 or 4 μM, overexpression of TMPRSS4 mRNA was observed in all cell lines (Supplementary Figure 2F). Pyrosequencing analysis (average of the 2 CpG analyzed) confirmed that the degree of TMPRSS4 promoter methylation was significantly decreased in most of the treated cell lines (Supplementary Figure 2G).

Taken together, all these results suggest that TMPRSS4 expression is controlled by promoter methylation, and that abnormal hypomethylation results in TMPRSS4 overexpression.

## DISCUSSION

Expression of the serine protease TMPRSS4 is highly increased in a variety of solid tumors and is associated with poor prognosis in some cancer types such as breast, colon, cervix, thyroid and liver [8, 11]. Our present study shows for the first time that TMPRSS4 is an independent prognostic indicator of reduced survival in patients with squamous NSCLC at early stages, thus validating our previous findings by mRNA analysis [7]. The protumorigenic and prometastatic role of TMPRSS4 described in experimental models may influence tumor features leading to worse clinical outcome. In particular, this protease induces EMT, cell motility and invasion in colon [12], lung [7, 20] and liver [11] cancer. Moreover, we have recently shown [14] that TMPRSS4 expression leads to the acquisition of a CSC phenotype and that levels in patients correlate with those of other CSC markers that predict poor prognosis, such as ALDH and OCT4 [21, 22].

Thus far, the molecular mechanisms that may explain why TMPRSS4 is highly expressed in lung tumors are largely underexplored. Hamamoto *et al.*, have shown that TMPRSS4 is upregulated in NSCLC by epigenetic silencing of the tissue factor pathway inhibitor-2 [23]. We now provide evidence showing epigenetic changes that elicit TMPRSS4 promoter hypomethylation in NSCLC. Moreover, we show that TMPRSS4 promoter methylation is inversely correlated with mRNA expression in both NSCLC patients and cell lines, which suggests that loss of methylation could constitute a main mechanism to express TMPRSS4. Although other molecular alterations might also influence expression of this protease in cancer, bioinformatic analyses using different public sources seem to indicate that genetic alterations of this protease are rare in NSCLC. Therefore, epigenetic deregulation is likely to be the main cause of TMPRSS4 overexpression in this tumor type.

Results from the 450k methylation array, and both bisulfite sequencing and pyrosequencing in our study show that, although TMPRSS4 promoter does not contain canonical CpG islands, there are relevant methylation regions in the TMPRSS4 promoter, which include CpGs located at positions −116 bp to +271 bp relative to the TSS. Analyses of ENCODE and transcription factor binding sites prediction databases (cREMaG and MotifScanner) suggest a possible involvement of transcription factors related to proliferation, EMT and inflammation (e.g. Zeb1, E2F1, Myc, NFkB and STAT3), whose binding in the vicinity of those CpGs could be mediated by hypomethylation. Future studies should determine whether binding of these factors is epigenetically regulated and whether this would have an effect on TMPRSS4 expression.

DNA hypomethylation is being increasingly acknowledged as a recurrent event in cancer, a mechanism that frequently affects oncogenes and tumor-promoting genes involved in proliferation, migration and metastasis [15]. In a genome-wide DNA methylation profiling study conducted in stage I NSCLC, authors described promoter hypermethylation in 496 CpGs and hypomethylation in 373 CpGs, which shows that frequency of both epigenetic modifications is similar [24]. Other tumor promoter genes described in NSCLC, such as ELMO3 and 14-3-3s, show similar patterns of promoter methylation/expression to those observed for TMPRSS4 [25, 26].

Our study shows that both TMPRSS4 hypomethylation and high protein expression predict shorter RFS in patients with SCC, but results are not so consistent for patients with ADC. Over the past few years, different studies have described genomic alterations associated with SCC that are under-represented in ADC, although targeted therapy based on these alterations is still to be developed [5]. Deciphering molecular mechanisms causative of tumor progression and metastasis will help establishing novel therapeutic options for patients with SCC tumors. In this regard, therapeutic approaches in TMPRSS4-expressing SCC tumors should be investigated in future studies. This could be achieved with small molecules, but based on our study, targeted restoration of promoter methylation may be an alternative therapeutic strategy. Epigenetic editing approaches are currently being investigated for specific modulation of gene expression. CRISPR/Cas9 and zinc fingers (ZF) editing methodologies have been used to modulate the activity of DNA methyltransferases (DNMTs) in a gene-specific way. For example, Stolzenburg *et al.*, have recently developed a biological tool that links ZF proteins with DNMT3A to methylate the DNA promoter of the SOX2 oncogene, which results in efficient gene expression silencing and antitumor effects *in vivo* [27].

In conclusion, we show for the first time that there is an epigenetic DNA hypomethylation of the TMPRSS4 promoter, a mechanism that may explain the TMPRSS4 overexpression found in NSCLC. We also demonstrate that both TMPRSS4 levels and promoter methylation status are useful biomarkers to predict poor prognosis in patients with lung SCC at early stages.

## MATERIALS AND METHODS

### Patients

To detect TMPRSS4 by immunohistochemistry, tissue microarrays (TMAs) containing samples from NSCLC (n=79) and matched non-malignant lung tissues (n=66) were used. Tumor samples were obtained from patients diagnosed at stage I or II who did not receive neoadjuvant therapy prior to surgery. Detailed histopathological and clinical characteristics of the patients are summarized in Supplementary Table 1. The TMAs included representative areas of the specimens with cores from each tumor at 3 different sites. Samples were obtained from patients who underwent tumor resection surgery at Clinica Universidad de Navarra (Pamplona, Spain). The study protocol was approved by the Institution and written informed consent was obtained for each patient. Immunostaining was carried out following standard procedures. For more details about immunohistochemistry, see Supplementary Materials and Methods (Suppl. M&M). Samples were blindly evaluated by two observers (M.R. and M.E.B.) considering both staining extension and intensity as previously described [28]. An H-score was established for each sample using those parameters. The median value was chosen as the cutoff to establish low and high TMPRSS4 expression levels.

For promoter methylation analysis, two cohorts of patients were used. We first evaluated our previously published FP7 CURELUNG discovery cohort of lung tumors (n=444 patients) and non-tumor lung tissue samples (n=25) [29] with the 450k methylation array to quantify the degree of methylation in each sample set. A subset of the aforementioned discovery cohort consisting in cancer tissues from 198 surgically resected NSCLC patients was also used for assessing the prognostic value of TMPRSS4 promoter methylation on relapse free survival. The clinical characteristics of these NSCLC patients are available in our previous publication [29]. A second cohort (validation cohort) was used to study TMPRSS4 promoter methylation status by pyrosequencing, which included 88 NSCLC samples from Clinica Universidad de Navarra. Characteristics of this cohort of patients are summarized in Supplementary Table 1.

This work followed REMARK (REporting recommendations for tumour MARKer prognostic studies) criteria for tumor marker studies.

### Cell lines, cluster analysis and treatment with a demethylating agent

A panel of 46 cell lines corresponding to different lung cancer histological types was included in this study (Supplementary Table 3). Of these, 16 cell lines representative of the 3 different methylation-based clusters identified in the 450k methylation array (see Results) were chosen for DNA methylation analysis by bisulfite genomic sequencing.

For treatments with 5-aza-2′deoxycitidine (Sigma, St. Louis, MO, USA), A549, H82, H23, H520 and H460 cells were cultured in RPMI and exposed to the drug at 2 μM and 4 μM concentrations. After 48h, cells were trypsinized and pellets were washed with PBS and maintained at −80°C for RNA and DNA extraction.

### DNA and RNA isolation and real time PCR

Genomic DNA was extracted from FFPET (formalin-fixed-paraffin-embedded tissue) using Cobas^®^ DNA Sample Preparation Kit, and RNA was obtained from frozen pellets using the Nucleospin RNA kit. Real time PCR was performed with SYBR green using standard protocols and primer sequences are shown in Supplementary Table 4. For detailed protocols see Suppl. M&M.

### DNA methylation analysis by bisulfite genomic sequencing

The Methyl Primer Express v1.0 software (Applied Biosystems) was used to identify the TMPRSS4 promoter CpGs and to design specific primers for the methylation analysis (Supplementary Table 4). None of the primers covered any CpG. Approximately 1 μg of DNA from each human lung cancer cell line was subjected to sodium bisulfite treatment using the EZ DNA Methylation-Gold kit (Zymo Research). A 394-bp fragment, −199 bp to +195 bp relative to the transcription start site (TSS), was amplified using 2 μL of bisulfite-converted DNA with Immolase Taq polymerase (Bioline) at 60°C for 35-40 cycles. The resulting PCR products were gel-purified (2% agarose) with NucleoSpin^®^ Gel and PCR Clean-up (Macherey-Nagel) and then cloned into the pGEMT Easy Vector System (Promega) according to the manufacturer's protocol. In all the samples, 10 colonies were randomly chosen, and DNA was purified using NucleoSpin^®^ 96 Plasmid (Macherey-Nagel) and sequenced. After sequencing analysis, results were transformed into percentages of CpGs showing methylation.

### DNA methylation analysis by bisulfite pyrosequencing

Quantitative DNA methylation analysis of the TMPRSS4 promoter was performed by bisulfite pyrosequencing of two consecutive potentially methylated cytosines located at −116 bp and −99 bp relative to the TSS. Bisulfite conversion of 500 ng of each DNA sample was performed with EZ DNA Methylation-Gold Kit (Zymo Research) according to the manufacturer's recommendations. Primer sequences (Supplementary Table 4) were designed with PyroMark Assay Design 2.0 (Qiagen). None of the PCR primers covered any CpG. PCR for TMPRSS4 promoter was performed with 1 μl of bisulfite converted DNA with biotinylated primers using an annealing temperature of 60°C and 50 cycles. PCR products were verified on 2% agarose gels before pyrosequencing analysis. Pyrosequencing was performed using a Pyro Gold SQA™ Reagent Kit (Qiagen) in a PyroMark Q96 System version 2.0.6 (Qiagen) according to the manufacturer's instructions. CpG site methylation quantification was obtained using Pyro Q-CpG 1.0.9 (Qiagen).

### Bioinformatic and statistical analysis

Different statistical methods were applied depending on the type of experimental data. The processed transcriptomic (RNA-Seq experiments) and DNA methylation data generated by the 450k methylation array for lung cancer datasets were downloaded from the public TCGA project portal [18]. Bioinformatic analysis was carried out with R and Bioconductor (freely available). The analysis of differential methylation patterns was performed with LIMMA (Linear Models for Microarray Data) [30]. Probes were selected as significant using the criteria of FDR<5%. The association between RNA-Seq expression levels and microarray methylation levels were tested with Pearson's correlation.

Correlation analysis between TMPRSS4 expression and promoter methylation in cell lines was assessed with Spearman's test and cells were clustered based on their methylation status by hierarchical clustering analysis using Euclidean distance and complete linkage analysis.

Normality of the data was assessed with the Shapiro-Wilk test. The association between TMPRSS4 expression and clinicopathological features of patients was analyzed by Pearson's chi-square test. Relapse free survival (RFS) and overall survival (OS) were evaluated with Kaplan-Meier curves and significant differences among groups were assessed by the logRank test. Overall survival was defined as the time from study enrollment to death. To evaluate the prognostic value of TMPRSS4, univariate and multivariate Cox proportional hazard analyses were used. To assess statistical differences between groups when comparing TMPRSS4 promoter methylation status in tumor versus non-malignant samples, the Mann-Whitney U test was used. Comparison of H-score for TMPRSS4 expression between non-malignant and malignant samples was assessed with T-test.

To classify patients with respect to their methylation levels in specific CpGs, we set a threshold β-value of 0.5 to define non-methylation (β<0.5) vs. methylation based on averaged 95^th^ percentile for control samples. Data was analyzed with the SPSS statistical software (version 17.0 for Windows SPSS) and GraphPad Prism 5 software (GraphPad). Statistical significance was defined as p<0.05 (*), p<0.01 (**), and p<0.001 (***).

## SUPPLEMENTARY MATERIALS AND METHODS



## Abbreviations

NSCLC: non-small cell lung cancer
SCC: squamous cell carcinoma
ADC: adenocarcinoma
TCGA: The Cancer Genome Atlas
TSS: Transcription Start Site
