# Supplementary material for: Epigenetic alterations leading to TMPRSS4 promoter hypomethylation and protein overexpression predict poor prognosis in squamous lung cancer patients
Source: Oncotarget. 2016 Mar 14;7(16):22752–69. doi: 10.18632/oncotarget.8045 (PMC5008398; doi:10.18632/oncotarget.8045)
Supplement: Supplementary file 1 [file oncotarget-07-22752-s001.pdf]

SUPPLEMENTARY MATERIALS AND METHODS

Immunohistochemistry for TMPRSS4

For immunostaining, slides were deparaffinized and rehydrated. Endogenous peroxidase was blocked with a 3% hydrogen peroxide solution and antigen retrieval was carried out by heating the samples in a microwave oven using citrate buffer (10 mM, pH 6). Tissues were then incubated overnight at 4ºC with anti-TMPRSS4 antibody (1:500 Ingenasa Inc, Madrid, Spain). Detection of primary antibody was performed with the Advance™ HRP system (Dako, Denmark) and peroxidase activity was developed with diaminobenzidine (Dako). Finally, slides were counterstained with haematoxylin, dehydrated and coverslipped with DPX mounting medium (VWR, Soulbury, UK).

DNA isolation

Briefly, samples were deparaffinized for 20 min at 60°C; immediately after, sections were immersed in xylene and absolute ethanol in consecutive steps and DNA was extracted according to the manufacturer's instructions. In the case of cell lines, NucleoSpin Tissue® kit (Macherey-Nagel) was used to extract DNA from frozen pellets. DNA concentrations were measured with Quant-it Picogreen dsDNA Assay Kit (Life Technologies).

RNA isolation and real time PCR

Purified RNA was obtained from frozen pellets with the Nucleospin RNA extraction kit (Macherey-Nagel) following manufacturer's instructions and reverse transcription was performed with PrimeScript kit (Takara). SYBR Green was used to carry out real time PCR and GAPDH was used as housekeeping gene.

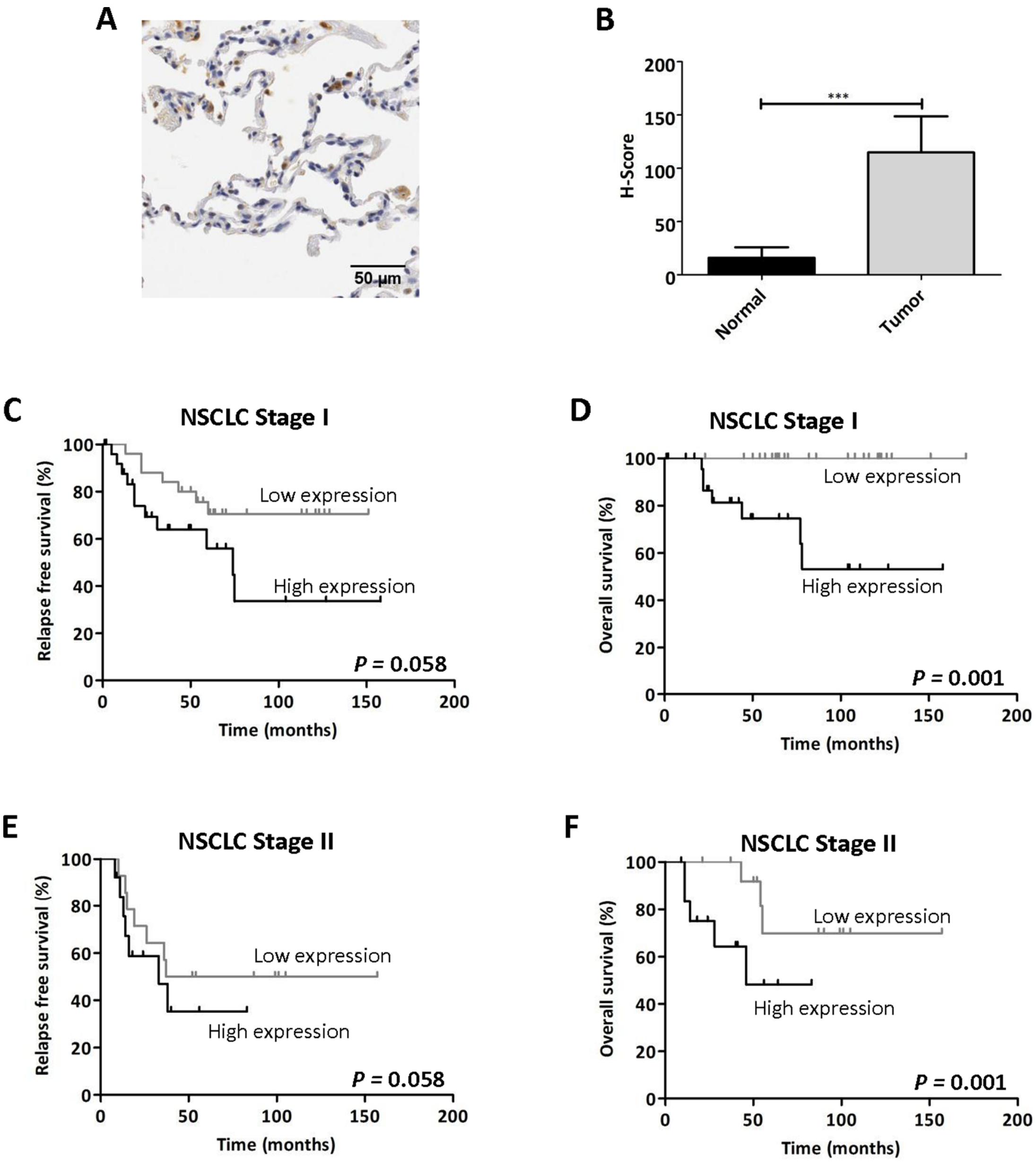

G

	RFS	OS
**All samples**	**0.001**	**<0.001**
**ADC**	0.052	**0.023**
**SCC**	**0.035**	**0.017**

Supplementary Figure S1: A. Representative image of non-malignant lung parenchyma immunostained with anti-TMPRSS4 antibody. Labeling is observed in some type II pneumocytes. **B.** Comparison of the H-score between non-malignant and tumor samples. **C-D.** Kaplan-Meier curves for stage I NSCLC patients (n=52) according to high or low TMPRSS4 protein expression. A significant reduction in OS is observed in patients with high TMPRSS4 (D). No statistical significance is reached in the case of RFS (C). **E-F.** Kaplan-Meier curves show similar results for stage II patients (n=27), regarding RFS (E) and OS (F). **G.** P-values corresponding to logRank tests after excluding patients that were treated with adjuvant chemotherapy/radiotherapy. Statistical differences show significant association between high TMPRSS4 levels and reduced RFS and OS. ***: p<0.001.

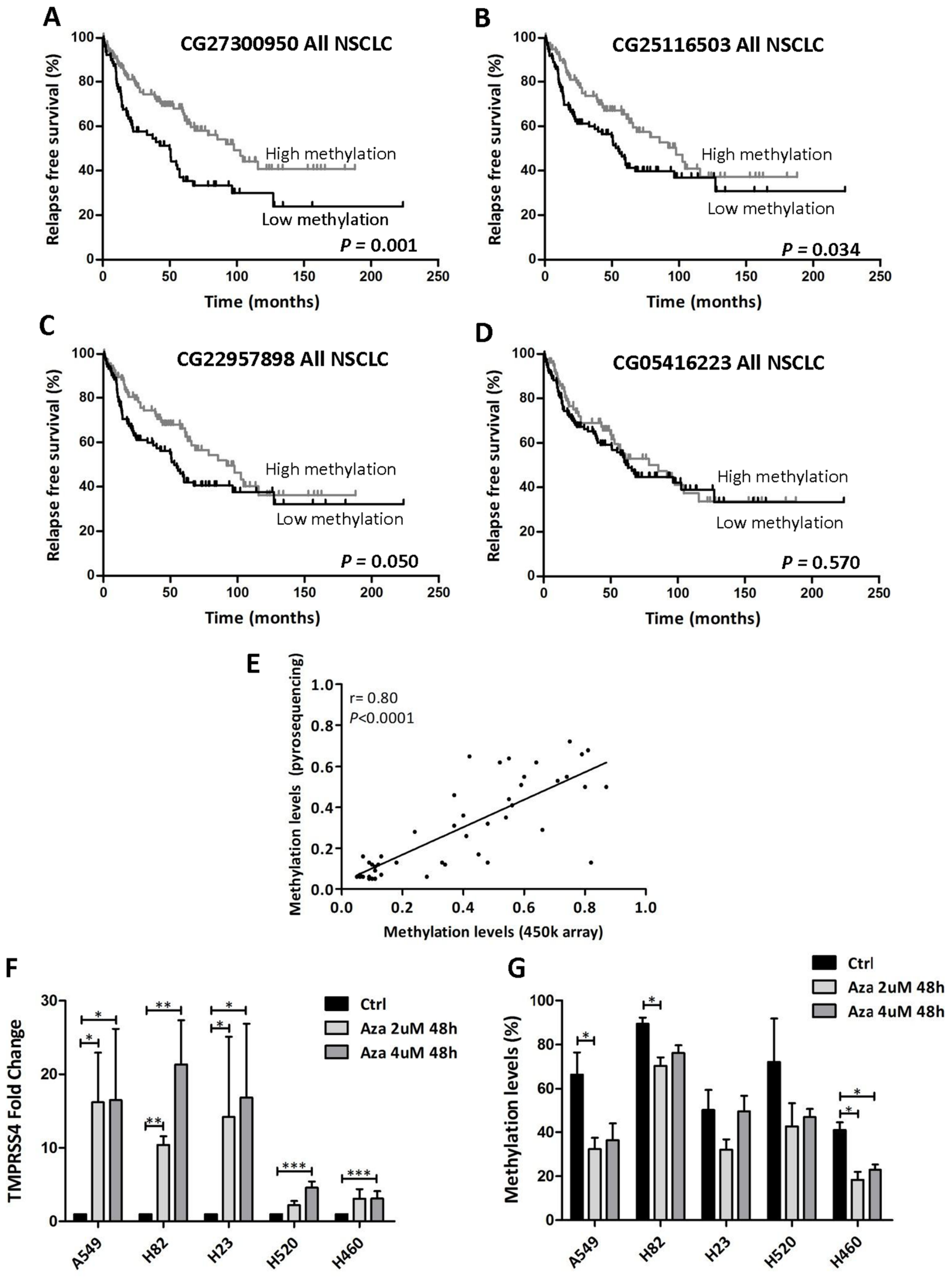

Supplementary Figure S2: A-D. RFS Kaplan-Meier curves for all NSCLC patients according to promoter methylation status for probes CG27300950 (A), CG25116503 (B), CG22957898 (C) and CG5416223 (D). **E.** A significant positive correlation is found between methylation status analyzed by pyrosequencing for probes CpGs -116 bp (CG03634928) and -99 bp (CG27300950) (average value of both CpGs) and methylation status from the 450K array. Pearson's correlation: r=0.80, p<0.0001. **F.** Overexpression of TMPRSS4 upon treatment with the demethylating agent 5-aza-2′-deoxycytidine (Aza) in lung cancer cells with low TMPRSS4 expression. Exposure to either 2 or 4 µM 5-aza-2′-deoxycytidine after 48h caused increased expression of TMPRSS4 in all the cells tested. **G.** Average values of methylation, obtained by pyrosequencing, in CpG sites -116 bp and -99 bp after treatment with Aza, showed decreased methylation levels in TMPRSS4.

Supplementary Table S1: Clinical and pathological characteristics of the patients

	TMA Cohort (n=79)	Validation Cohort (n=88)
	**n (%)**	**n (%)**
Age (median ± SD)	65±10.1	65±8.5
**Gender**		
Female	12 (15.2)	13 (14.8)
Male	67 (84.8)	75 (85.2)
**Smoking status**		
Never smoker	8 (10.1)	9 (10.2)
Former smoker	53 (67.1)	57 (64.8)
Current smoker	18 (22.8)	22 (25.0)
**Histology**		
ADC	39 (49.4)	28 (31.8)
SCC	33 (41.8)	52 (59.1)
Others	7 (8.9)	8 (9.1)
**Stage**		
I	52 (65.8)	47 (53.4)
II	27 (34.2)	28 (31.8)
III		11 (12.5)
IV		1 (1.1)
Missing		1 (1.1)
**Grade**		
Well	9 (11.4)	8 (9.1)
Moderately	33 (41.8)	42 (47.7)
Poorly	30 (38.0)	35 (39.8)
Missing	7 (8.9)	3 (3.4)
**pT**		
T1	31 (39.2)	37 (42.0)
T2	43 (54.4)	39 (44.3)
T3	5 (6.3)	10 (11.4)
T4		1 (1.1)
Missing		1 (1.1)
**pN**		
N0	65 (82.3)	64 (72.7)
N1	14 (17.7)	16 (18.2)
N2		7 (8.0)
Missing		1 (1.1)

pN = pathological N stage; pT = pathological T stage.

Supplementary Table S2: Relationship between TMPRSS4 expression and clinicopathological characteristics of NSCLC patients

	**NSCLC (n=79)**			**ADC (n=39)**			**SCC (n=33)**		
	**n (%)**	**n (%)**		**n (%)**	**n (%)**		**n (%)**	**n (%)**	
	**Low**	**High**	***P***	**Low**	**High**	***P***	**Low**	**High**	***P***
**Age**									
≤65	24 (57.1)	18 (42.9)	0.141	16 (69.6)	7 (30.4)	0.394	8 (50.0)	8 (50.0)	0.226
>65	15 (40.5)	22 (59.5)		9 (56.3)	7 (43.8)		5 (29.4)	12 (70.6)	
**Gender**									
Female	7 (58.3)	5 (41.7)	0.500	6 (66.7)	3 (33.3)	0.855	1 (50.0)	1 (50.0)	0.751
Male	32 (47.8)	35 (52.2)		19 (63.3)	11 (36.7)		12 (38.7)	19 (61.3)	
**Smoking status**									
Never smoker	4 (50.0)	4 (50.0)		4 (57.1)	3 (42.9)				
Former smoker	29 (54.7)	24 (45.3)	0.292	19 (70.4)	8 (29.6)	0.392	9 (42.9)	12 (57.1)	0.590
Current smoker	6 (33.3)	12 (66.7)		2 (40.0)	3 (60.0)		4 (33.3)	8 (66.7)	
**Histology**									
ADC	25 (64.1)	14 (35.9)							
SCC	13 (39.4)	20 (60.6)	**0.017**						
Others	1 (14.3)	6 (85.7)							
**Stage**									
I	25 (48.1)	27 (51.9)	0.750	15 (62.5)	9 (37.5)	0.792	10 (40.0)	15 (60.0)	0.900
II	14 (51.9)	13 (48.1)		10 (66.7)	5 (33.3)		3 (37.5)	5 (62.5)	
**Grade**									
WD/MD	21 (50.0)	21 (50.0)	0.576	14 (66.7)	7 (33.3)	0.455	7 (36.8)	12 (63.2)	0.727
PD	13 (43.3)	17 (56.7)		7 (53.8)	6 (46.2)		6 (42.9)	8 (57.1)	
**pT**									
T1	17 (54.8)	14 (45.2)	0.434	13 (81.3)	3 (18.8)	0.063	4 (28.6)	10 (71.4)	0.275
T2/T3	22 (45.8)	26 (54.2)		12 (52.2)	11 (47.8)		9 (47.4)	10 (52.6)	
**pN**									
N0	32 (49.2)	33 (50.8)	0.958	20 (64.5)	11 (35.5)	0.916	12 (41.4)	17 (58.6)	0.530
N1	7 (50.0)	7 (50.0)		5 (62.5)	3 (37.5)		1 (25.0)	3 (75.0)	

WD = well differentiated; MD = moderately differentiated; PD = poorly differentiated.

Supplementary Table S3: Cell lines and their corresponding histological type used in the study

Cell line	Type	Cell line	Type
COR-L88	Small cell carcinoma	H2009	Adenocarcinoma
H1436	Small cell carcinoma	H2087	Adenocarcinoma
H187	Small cell carcinoma	H2126	Adenocarcinoma
H209	Small cell carcinoma	H2228	Adenocarcinoma
H345	Small cell carcinoma	H23	Adenocarcinoma
H446	Small cell carcinoma	H322	Adenocarcinoma
H510	Small cell carcinoma	H358	Adenocarcinoma
H69	Small cell carcinoma	H441	Adenocarcinoma
H82	Small cell carcinoma	H650	Adenocarcinoma
N417	Small cell carcinoma	HCC-44	Adenocarcinoma
103H	Large cell carcinoma	HCC-827	Adenocarcinoma
97TM1	Large cell carcinoma	LXF-289	Adenocarcinoma
H1299	Large cell carcinoma	PC-14	Adenocarcinoma
H460	Large cell carcinoma	H1703	Squamous cell carcinoma
H661	Large cell carcinoma	H1869	Squamous cell carcinoma
A549	Adenocarcinoma	H2170	Squamous cell carcinoma
H1395	Adenocarcinoma	H226	Squamous cell carcinoma
H1437	Adenocarcinoma	H520	Squamous cell carcinoma
H1568	Adenocarcinoma	HCC-15	Squamous cell carcinoma
H1648	Adenocarcinoma	NH91	Squamous cell carcinoma
H1650	Adenocarcinoma	SW900	Squamous cell carcinoma
H1792	Adenocarcinoma	HCC-366	Adenosquamous
H1975	Adenocarcinoma	H727	Carcinoid

Supplementary Table 4: Primers used in this study

Primers	Sequence
**Real time RT-PCR**	
TMPRSS4 forward	GGTCAGCATCCAGTACGACA
TMPRSS4 reverse	GCACCTTCCAGTTGAACACA
GADPH forward	ACTTTGTCAAGCTCATTTCC
GADPH reverse	CACAGGGTACTTTATTGATG
**Bisulfite genomic sequencing**	
TMPRSS4 forward	GTATTTAGAAGGTAGGGGGAGG
TMPRSS4 reverse	CAACAAATCAAAAATCCCTAATC
**Pyrosequencing**	
TMPRSS4 forward	AGTTAATATTTAGTTGGGTGGAAGT
TMPRSS4 reverse	[Btn]AAACAACCCTACAAATAAAATTACCA
TMPRSS4 sequence	GTTGGGTGGAAGTTATTTA

[Btn] = biotinylated.
